# Impact of Fluorine in Manganese Citrate Synthesis on Structure and Value-Added Decomposition Products

**DOI:** 10.3390/molecules30081794

**Published:** 2025-04-16

**Authors:** Aljaž Škrjanc, Emanuela Trstenjak, Mojca Opresnik, Matej Gabrijelčič, Amalija Golobič, Nataša Zabukovec Logar

**Affiliations:** 1 National Institute of Chemistry, Hajdrihova 19, SI-1000 Ljubljana, Slovenia; aljaz.skrjanc@ki.si (A.Š.); emanuela.trstenjak@silkem.si (E.T.); mojca.opresnik@ki.si (M.O.); matej.gabrijelcic@ki.si (M.G.); 2 School of Science, University of Nova Gorica, Vipavska 13, SI-5000 Nova Gorica, Slovenia; 3 Faculty of Mathematics and Physics, University of Ljubljana, Jadranska Ulica 19, SI-1000 Ljubljana, Slovenia; 4 Faculty of Chemistry and Chemical Technology, University of Ljubljana, Večna Pot 113, SI-1000 Ljubljana, Slovenia; amalija.golobic@fkkt.uni-lj.si

**Keywords:** manganese(II) carboxylate, single-crystal structure determination, metal–organic structure, porous manganese oxides, porous manganese carbons

## Abstract

Synthesis of manganese citrates was carried out in the presence and absence of fluoride ions. The presence of HF or NaF led to the formation of new layered manganese citrates whose crystal structures were determined by single-crystal X-ray diffraction. The prepared single-phase citrates with and without fluoride ions were then used as precursors for the preparation of mesoporous Mn_x_O_y_ and hierarchically porous Mn_x_O_y_@C phases, which could be of interest for catalytic, sorption, and electrochemical applications.

## 1. Introduction

Manganese carboxylate complexes have long been of special interest since they are known to be active in some metalloenzymes and proteins [1,2]. On the other hand, manganese-based inorganic catalysts for the treatment of organic pollutants in the environment have also been well established and studied [3,4,5,6,7,8,9]. Metal–organic frameworks (MOFs) with crystalline three-dimensional porous structures perfectly combine the characteristics of inorganic constituents with those of organic ligands and are readily used for gas capture and separation applications [10]. While MOFs also show promise in catalysis, an issue still remains in the limitation of MOFs for thermal and photothermal catalysis due to their finite thermal stability. A recently proposed approach to the use of MOFs as precursors for value-added thermal decomposition products has received a lot of attention [11,12,13]. In addition to the preparation of MOF-derived catalysts [14], MOF-derived supercapacitors [15] and electrode materials [16,17,18,19,20] have also been reported. Namely, the selected thermal treatment of MOF precursors can lead to the formation of porous metal oxides and metal(oxide)–carbon composites, adding to the performance in the above-listed applications.

In light of the advances in coordination chemistry and due to the boom of research into MOFs, we decided to revisit Mn-based metal–organic materials synthesis [21,22,23,24,25,26,27]. A recently reported Co-citrate complex for catalytic application [28,29] motivated us to re-examine the potential of citric acid in the formation of new manganese carboxylate structures. Citric acid, an α-hydroxyl tricarboxylic acid, and citrate ions are simple and easily accessible reagents. A literature review revealed a few known manganese citrate complexes, [Mn(H_2_O)_2_][Mn_2_(HCit)_2_(H_2_O)_4_]^.^4H_2_O [30] and (NH_4_)_4_[Mn(HCit)_2_] [31], two chain-like coordination structures, [Mn(C_6_H_6_O_7_)(H_2_O)] [32] and [Mn(H_2_O)_6_][Mn(HCit)(H_2_O)]_2_^.^2H_2_O [33], a layered structure, {K[Mn(HCit)(H_2_O)]} [34], as well as quite a few three-dimensional framework structures, i.e., [{Mn(HCit)(H_2_O)_2_}_2_Mn(H_2_O)_4_], [MnNa(HCit)], [MnCa_2_(HCit)_2_(H_2_O)_4_], [Mn(HCit)(H_2_O)], and [{Mn(HCit)(H_2_O)_2_}_2_Mn(H_2_O)_2_] [35].

Here, we report a hydrothermal synthesis of two new manganese citrate complexes, which were prepared in fluoride media. In recent years, fluorine incorporation has shown tremendous promise in both the fields of materials science [36] and proteomics [37]. As such, we decided to test the synthesis in the presence of HF and NaF, as fluorine ions are known structure-directing agents and mineralizers, enhancing crystal formation and growth [38]. In the fluoride media, two new layered manganese citrate compounds were prepared, their crystal structures solved, and thermal behavior determined. While HF has already been used to form defects in Mn-based MOFs, partially replacing benzenedicarboxylate ligands [39], this is to our knowledge the first report on the use of fluorine ions in the preparation of new manganese citrate phases. Both, the new citrates, as well as a benchmark fluorine-free citrate, were then calcined in both air and argon, and the thusly prepared value-added oxides and Mn_x_O_y_@C textural properties were evaluated.

## 2. Results and Discussion

### 2.1. Synthesis

The PXRD data for fluorine-free product revealed that the obtained compound was a known manganese citrate with a crystal structure that was already reported in the literature, i.e., chain Mn complex structure [Mn_2_(HCit)(H_2_O)] (MnCit CCDC:WADHAL) [32]. As shown in the Rietveld plot in Figure S1, the measured PXRD of fluorine-free product is in agreement with the calculated diffraction pattern of WADHAL.

**Figure 1:**
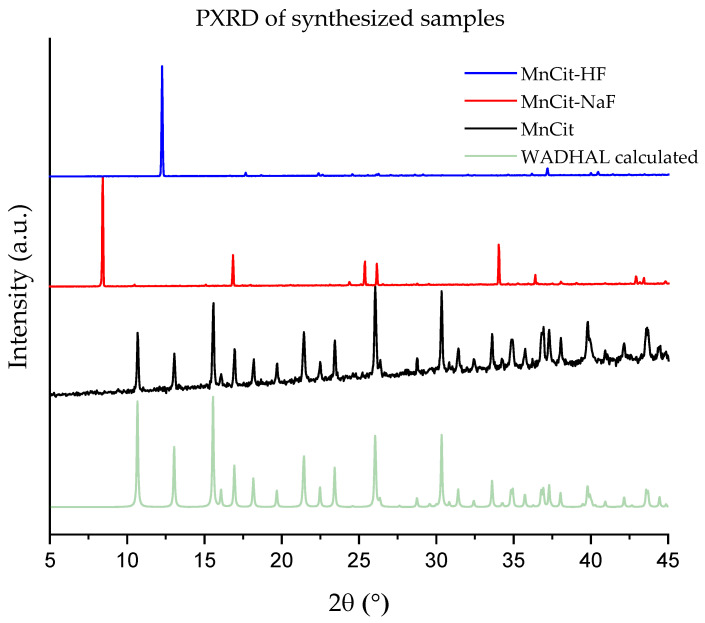
PXRD of obtained phases of manganese citrates and theoretical pattern for MnCit (WADHAL).

The PXRD (Figure 1) data for both fluorine-based products revealed two unknown crystalline phases. When only one source of fluorine was used, single-phase large crystallites suitable for single-crystal X-ray structure determination could be obtained as was confirmed by the Rietveld plots in Figures S2 and S3. A 50/50 molar ratio of fluorine sources led to a mixed-phase system of the two new phases.

The optimal solvent system was determined to be ethanol/water with a small addition of HF. In other cases, when more HF or ethanol was present, additional phases of MnF_2_ and/or MnCit were formed. The temperature and time of crystallization had some impact on the morphology of the crystals, but not on the phase composition in the final product. In the case of the NaF synthesis, changing the Mn/NaF ratio led to the formation of products with lower crystallinity or a MnCIT-NaF and MnCIT mixed-phase system.

### 2.2. Crystal Structures

Single-crystal X-ray diffraction analysis revealed two layered manganese(II) carboxylate structures, MnCit-HF and MnCit-NaF. The atomic parameters of both crystal structures are presented in Table S2, with bond distances and angles in Table S3. The +2 oxidation state of manganese was also confirmed by bond valence sum calculations, BVS (Table S5).

**Figure 2:**
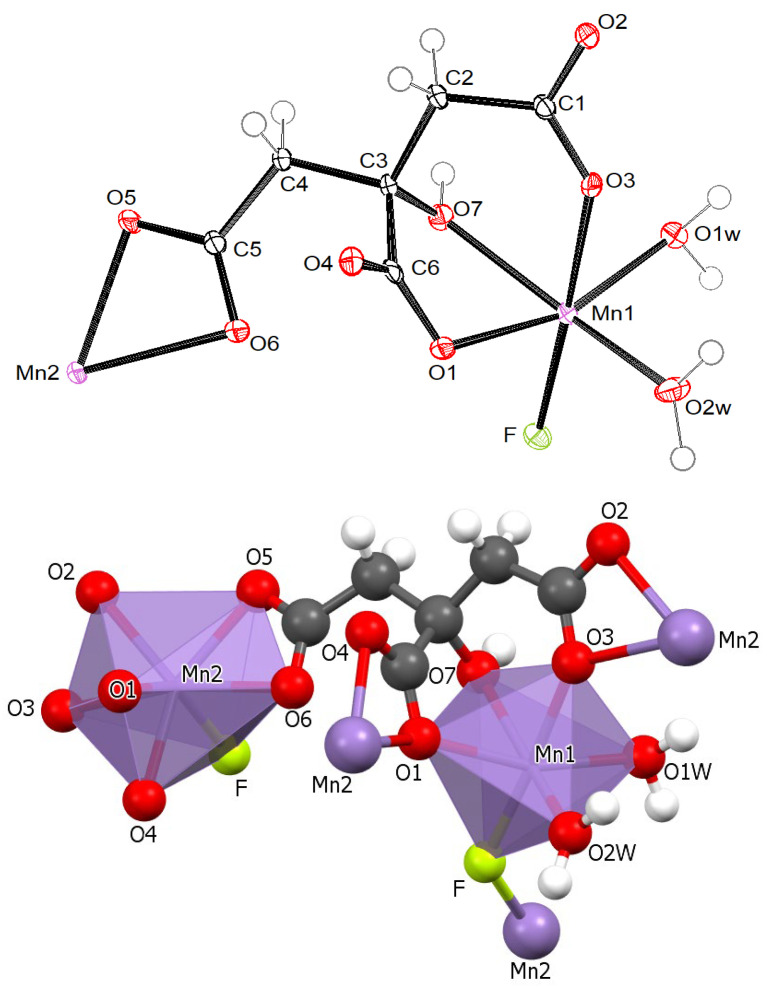
(**top**) The asymmetric unit of MnCit-HF with labeling of non-hydrogen atoms. Ellipsoids are drawn on a 50% probability level; (**bottom**) the coordination of Mn(1) and Mn(2) atoms in MnCit-HF (**bottom**).

The ORTEP drawing of the asymmetric unit of MnCit-HF with the formula [Mn_2_(C_6_H_5_O_7_)(H_2_O)_2_F] is shown in Figure 2 (top). There are two different crystallographic manganese sites of manganese(II) central ions, Mn(1) and Mn(2). As shown in Figure 2 (bottom), the Mn(1) ion has a slightly distorted octahedral coordination composed of five oxygen atoms and one fluorine atom. The Mn(2) ion has a distorted pentagonal bipyramidal geometry constructed of six oxygen atoms and one fluorine atom. Mn(1) and Mn(2) are interconnected by a bridge of citrate and fluoride ligands. Each citrate ion is, via all its seven O atoms, coordinated to four manganese(II) central ions.

**Figure 3:**
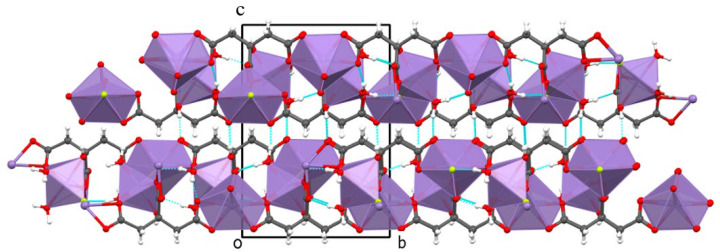
View of the MnCit-HF structure along the *a*-axis. Hydrogen bonds are colored light blue.

Polyhedrons of Mn(2) and Mn(1)-octahedrons are then interconnected through the F linkers into a layer parallel with the *ab* plane. Layers are stacked along the *c*-axis (Figure 3). The structure is stabilized by O-H…O and O-H…F hydrogen bonds. The hydrogen-bonding geometry (Å, °) is summarized in Table S4. The number and orientation of H-bonds confirm the proposed structural model, with terminal aqua and bridging fluoride ligands.

**Figure 4:**
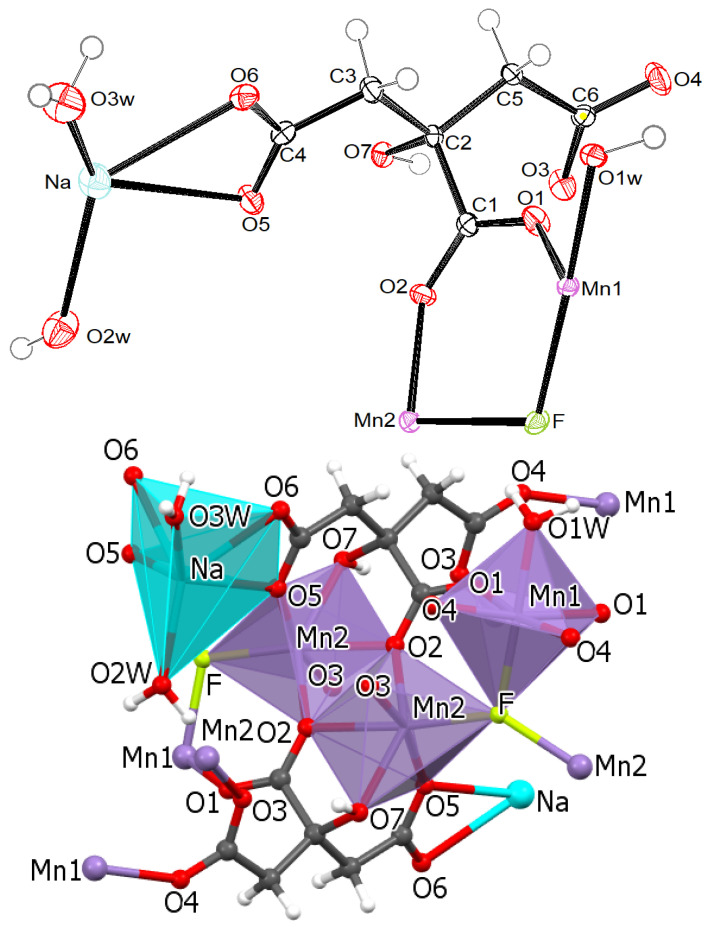
(**top**) The asymmetric unit of MnCit-NaF with labeling of non-hydrogen atoms. Ellipsoids are drawn on 50% probability: (**bottom**) the coordination of Mn1, Mn2, and Na atoms in MnCit-NaF.

**Figure 5:**
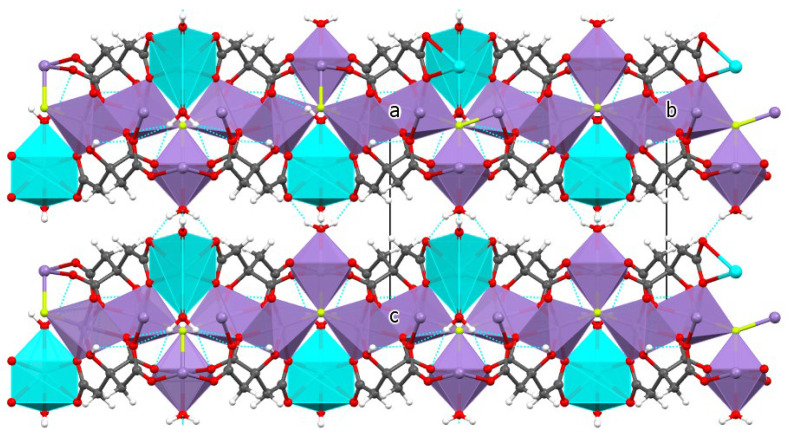
View of the MnCit-NaF structure along the *a*-axis. Hydrogen bonds are presented in light blue.

The formula of MnCit-NaF is Na(H_2_O)_2_[Mn_3_(C_6_H_5_O_7_)_2_(H_2_O)F]. The asymmetric unit is presented in Figure 4. Several atoms lie in special positions: Na, O2w, O3w, H1w3, and H2w3 lie on the same mirror plane, while Mn1, F, and O1w lie on another, symmetry-related mirror plane. Both manganese (II) central ions, Mn(1) and Mn(2) are in an octahedral coordination environment (Figure 4). Sodium cations are coordinated by four oxygen atoms from two citrate ions and by two water molecules. The coordination polyhedron is a distorted trigonal prism. The fluoride ion is part of the coordination environment of Mn octahedra and is bonded to three Mn atoms. The two Mn(2) octahedra share one edge. Each citrate ion is coordinated to a sodium cation via two O atoms and to four manganese(II) cations via six O atoms—O5 is bonded to Na and Mn(2). With all these connections, layers parallel to the *ab* plane are formed. As is shown in Figure 5, such planes are stacked along the *c*-axis. Layers are connected by O-H…O intermolecular hydrogen bonds between water molecules from neighboring planes. The geometric parameters of hydrogen bonds are given in Table S4.

In hopes of gaining further structural information about the fluorine in the structures, MAS NMR measurements were attempted for both ^19^F and ^23^Na (Section S2). In the ^23^Na spectra of the MnCIT-NaF sample, we could observe one main contribution at around −20 ppm, with a very small contribution at around 20 ppm; the second contribution is most likely some small Na impurity not removed by the washing step. The ^19^F spectra of both appear to only have one contribution, which can be attributed to the PTFE that the rotors are made of. A reference MnF_3_ sample was recorded under the same conditions, and the main peak of pure MnF_3_ (−122 ppm) overlaps greatly with the PTFE signal. The overlap in peak positions in combination with the seemingly larger peak broadening than expected leads to the samples being not suitable for ^19^F ssNMR study.

### 2.3. Analysis of Thermal Properties

**Figure 6:**
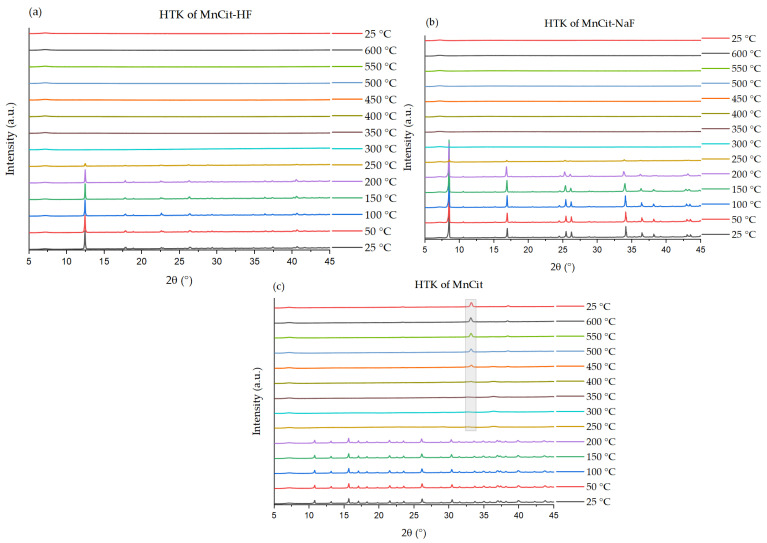
X-ray thermodiffractogram of (**a**) MnCit-HF, (**b**) MnCit-NaF, and (**c**) MnCit. In (**c**), the main peak of the formed crystalline Mn_2_O_3_ is highlighted.

Variable-temperature powder X-ray diffraction (HTXRD) was used to investigate the thermal stability of the three citrates (Figure 6). All three materials studied retain their crystallinity up to 200 °C, with both fluorine phases showing some residual diffraction at 250 °C despite a significant loss of crystallinity. The observed structural stability is in line with TGA-observed mass loss (Figure S5). In general, it appears that the incorporation of fluorine did not significantly affect the thermal stability of the Mn citrates. Only in the case of MnCit was a crystalline Mn_2_O_3_ phase observed after thermal decomposition; in the fluorinated samples, the only peak observed was the broad peak at around 7 °2θ, which belongs to the sample holder. We anticipate that the nature of the layered structures of both fluorine samples may lead to slower oxide formation kinetics, compared to the chain-like fluoride-free sample, which is why no crystalline oxide formation was observed in the HTXRD.

### 2.4. Mesoporous Oxide Formation

**Figure 7:**
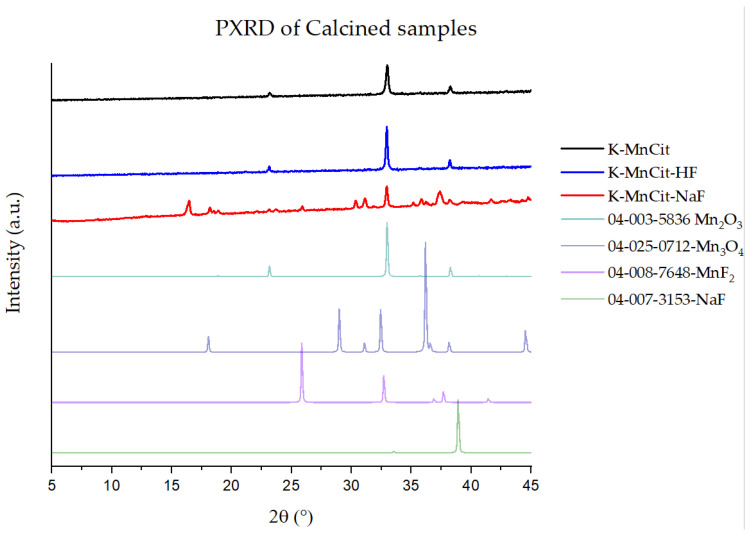
PXRD of calcined samples, with calculated reference diffractograms from PDF cards of identified phases.

With HTXRD showing that the formation of Mn_2_O_3_ starts at around 400 °C and that the peaks narrow up to 550 °C, we decided to calcine our samples in air at 550 °C. The products successfully calcined in air were denoted with K-. PXRD analysis (Figure 7) showed that Mn_2_O_3_ was obtained in the case of MnCit and MnCit-HF_._ The MnCit-NaF sample showed a lot of additional peaks, some of which we could assign to MnF_2_, NaF, and Mn_3_O_4_. The presence of additional phases can be potentially explained due to the presence of Na in the structure and possibly due to shorter distances between Mn in MnCit-NaF compared to the other two phases.

**Figure 8:**
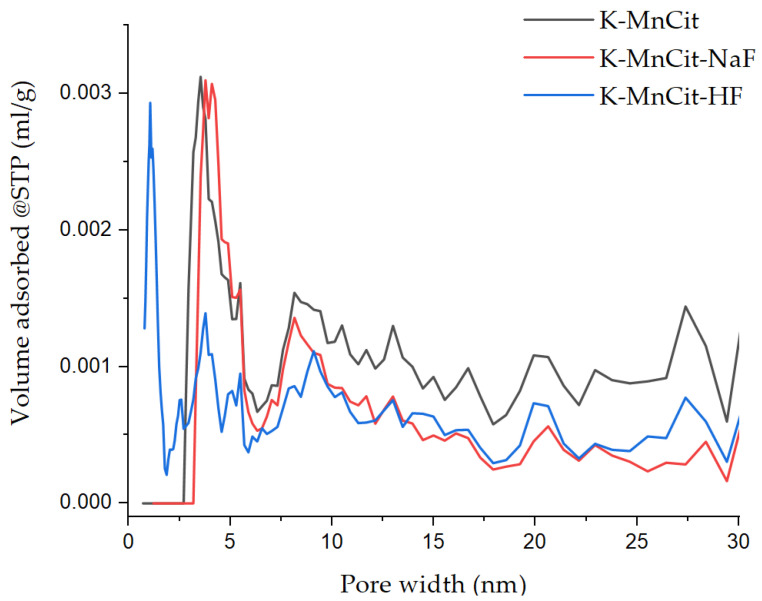
N_2_ physisorption determined pore size distribution.

N_2_ physisorption experiments (Section S3) were then performed on the samples to determine the textural properties of obtained pure oxides. K-MnCit and K-MnCit-HF had S_BET_ values of 19 m^2^/g and 15 m^2^/g, respectively. Modeling of the pore size distribution (Figure 8), in combination with the use of the t-plot method, reveals that while both had apparent microporosity, it was very low, with both samples exhibiting micropore volumes of 0.001 mL/g (K-MnCit) and 0.002 mL/g (K-MnCit-HF). K-MnCit-NaF had the lowest S_BET_ of 14 m^2^/g and the same micropore volume as K-MnCit. Looking at the total pore volume, we see that MnCit, with 0.14 mL/g, had almost triple the total pore volume of K-MnCit-HF, which had a total pore volume of 0.048 mL/g, while K-MnCit-NaF had the lowest total pore volume, with 0.033 mL/g. All prepared oxides had comparable total pore volumes to the ones prepared from Mn-MIL-100 [14], with all samples showing at least double the surface area of the 3D MOF-derived samples.

**Figure 9:**
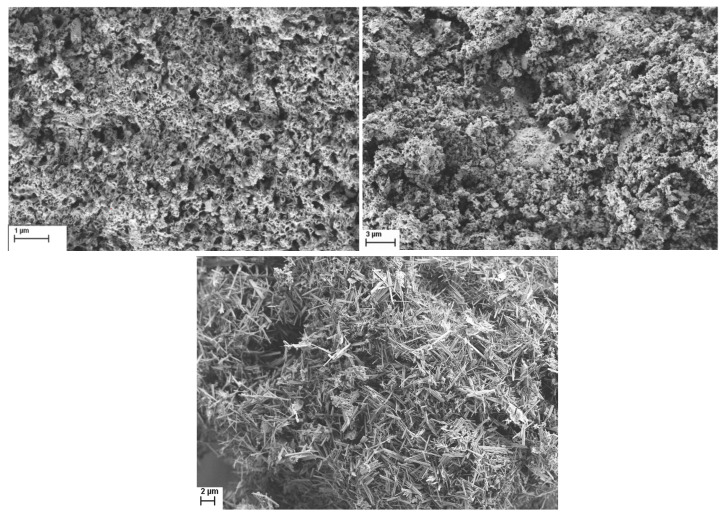
SEM images of Mn citrate-derived oxides, K-MnCit, K-MnCit-HF, and K-MnCit-NaF.

SEM imaging (Figure 9) showed no significant differences between the two Na-free samples, with both showing agglomerated particles. The outlier was K-MnCit-NaF, where SEM images showed distinct needle-like particles, in accordance with the PXRD results, where multiple phases were determined.

### 2.5. Carbonization of the Samples

**Figure 10:**
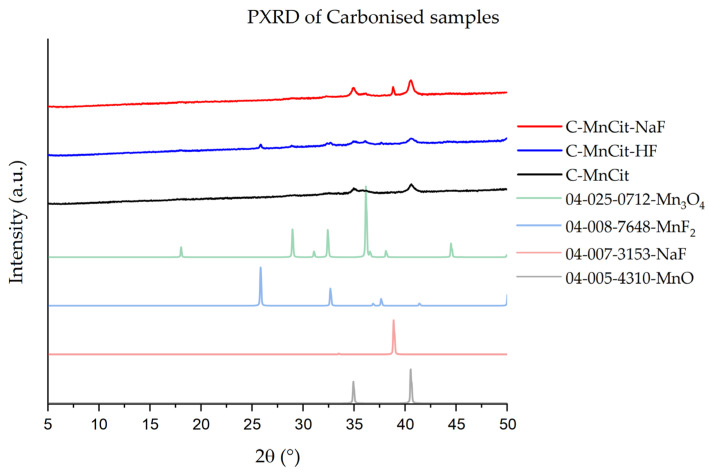
PXRDs of carbonized samples; the reference diffractograms were obtained from the individual PDF cards.

**Figure 11:**
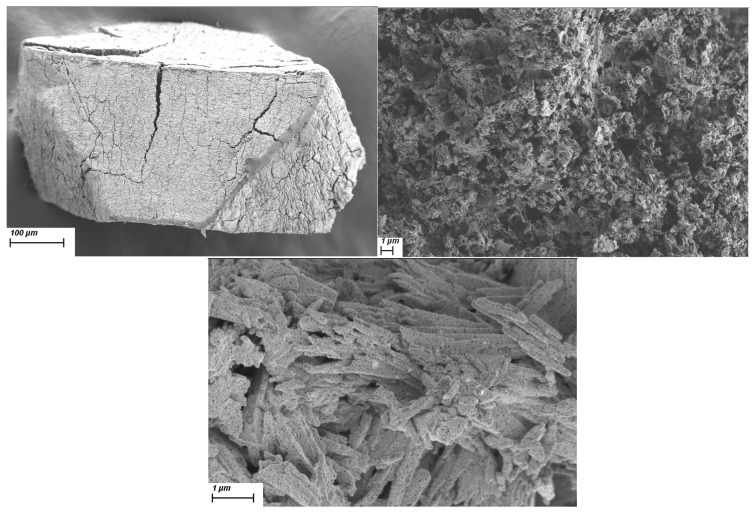
SEM images of carbonized samples: C-MnCit-HF (**top left**), C-MnCit-NaF (**top right**), and C-MnCit (**bottom**).

We then decided to also test out the prepared materials for the preparation of porous carbon with metal oxide particles. This was achieved through carbonization of the samples, denoted with C-. An additional 50 °C increase in thermal treatment temperature in Ar was used, just in case, to hopefully achieve single-phase oxide products on porous carbon. PXRD analysis (Figure 10) of the prepared carbonized samples shows that in all cases, MnO was formed as the main product. A pure MnO@C product was determined only for C-MnCit. In the case of the other two, some additional phases, i.e., MnF_2_, Mn_3_O_4_, and NaF, were present in the PXRD. The SEM images (Figure 11) of C-MnCit-HF show that very large particles of parent-MnCit materials were retained. The C-MnCit sample revealed needle-like particles on which multiple small crystallites could be observed, while the C-MnCit-NaF samples seemed to contain the least uniform particles, with overall smaller particles.

For the prepared materials, the N_2_ isotherms were collected (Section S3), and this time, we observed the same trend in porosity. C-MnCit-HF had higher microporosity and a slightly larger surface area at 83 m^2^/g, while the C-MnCit sample had a slightly higher S_BET_ of 99 m^2^/g. The C-MnCit-NaF sample had a S_BET_ of 78 m^2^/g. What we can observe is a switch in the trend of the oxides. Here, the highest V_total_ was determined for C-MnCit-HF (0.14 mL/g), followed by C-MnCit-NaF (0.097 mL/g) and finally C-MnCit (0.07 mL/g). Similarly, C-MnCit had the highest micropore volume (0.024 mL/g), followed by C-MnCit-HF (0.012 mL/g), while C-MnCit-NaF exhibited almost no microporosity (0.006 mL/g). EDS mapping was performed to ensure homogeneity of the Mn distribution; in all three samples, Mn was found to be homogenously dispersed throughout the sample (Section S4).

Both sodium-free samples therefore led to comparable hierarchically porous carbon supports with homogenously incorporated oxide nanoparticles. As such, they can potentially be suitable for various catalytic applications [15,20].

## 3. Materials and Methods

### 3.1. Materials

Manganese(II) acetate tetrahydrate (Mn(AcO)_2_.4H_2_O, 99%, Fluka, Dorset, UK), citric acid (HCit (99.8%, Baker, Den Helder, NL, USA), hydrofluoric acid—HF (40%, Merck, Darmstadt, Germany), sodium fluoride (NaF), and ethanol (EtOH, 97%) were obtained commercially. Water was deionized in-house.

### 3.2. Synthesis

Synthesis of the products was performed using reaction gels with the following molar ratios of reactants: 1 Mn(CH_3_COO)_2_.4 H_2_O : 1 C_6_H_8_O_7_ : 56 H_2_O : 43 C_2_H_5_OH : 0.5 XF (for HF and NaF synthesis). Manganese(II) acetate tetrahydrate (2.45 g, 0.01 mol) was dissolved in water (10.08 g, 0.56 mol). Citric acid (1.92 g, 0.01 mol) was dissolved in ethanol (19.78 g, 0.43 mol), separately. Both solutions were merged together and stirred for 5 min at room temperature. For the HF-based synthesis, at this stage, HF (0.26 g 40% HF, 0.005 mol) was added dropwise to the reaction mixture. For the NaF synthesis, anhydrous salt (0.21 g, 0.005 mol) was added to the Mn precursor solution.

The final gels were moved into Teflon-lined autoclaves (50 mL) and heated at 150 °C for 5 days. After cooling to room temperature, the products were filtered, washed with deionized water, and dried in a ventilation oven at 50 °C. The products were named MnCit-HT and MnCit-NaF, respectively. In the case of HF synthesis, a small amount of white powder, attributed to MnCit impurities, was removed from the pink crystals by washing in an ultrasound bath.

For MnCit synthesis, the fluorine source was omitted, due to a lack of formation of large crystal particles, and the ultrasound washing step was omitted.

The synthesized layered manganese citrates were then calcined in airflow at 550 °C for five hours with a heating rate of 10 °C/min. The products were named K-MnCit, K-MnCit-HT, and K-MnCit-NaF.

Carbonization of the synthesized manganese citrates was carried out at 600 °C for five hours in Ar with a heating rate of 2 °C/min. The products were named C-MnCit, C-MnCit-HT, and C-MnCit-NaF.

### 3.3. Characterization

The obtained products were checked for crystallinity and phase purity by collecting powder X-ray diffraction data (PXRD) on a PANanalytical X’Pert PRO high-resolution diffractometer (Malvern Panalytical, Malvern, UK) using CuKα1 radiation (1.5406 Å) in the 2θ range from 3 to 50° (100 s per step 0.017° 2θ) with a fully opened X’Celerator detector. The diffractograms were analyzed with the HighScore Plus 4.9 program package (Malvern Panalytical, Malvern, UK). Characterization and purity of single-phase samples of MnCit, MnCit-HF, and MnCit-NaF were confirmed by Rietveld refinement, which was performed with the Topas2.1 program (Bruker AXS, Karlsruhe, Germany). We have refined the scale factor, zero error, background, crystal size, strain parameters, and unit cell parameters.

The size, form, and morphology of the crystals were studied with a SUPRA 35 VP scanning electron microscope (SEM) (Carl Zeiss, Jena, Germany) and an Aztec energy dispersion spectrometer (Oxford Instruments, Oxford, UK). An energy-dispersive X-ray microanalyzer (EDX) connected to a scanning electron microscope was used to determine the amount of manganese and carbon in gold-coated samples.

Single-crystal diffraction data of MnCit-HF and MnCit-NaF compounds were collected using MoKα radiation and omega scans at 150.0(1) K on an Agilent SuperNova dual-source diffractometer with an Atlas detector and mirror monochromator (Agilent, Santa Clara, CA USA). The data were processed using CrysAlisPro 2024 version 1.171.43.121a (Rigaku Oxford Diffraction, Yarnton, UK), with multi-scan empirical absorption correction. Structures were solved by direct methods using SIR97 [40]. A full-matrix, least-squares refinement on F^2^ was employed with anisotropic temperature–displacement parameters for the non-hydrogen atoms. H atoms in all structures were observed in difference Fourier maps. Nevertheless, H atoms from methylene groups were placed at calculated positions and treated as riding. H atoms bonded to oxygen atoms were located in the difference Fourier maps. For the refinement of the positions of H atoms from water molecules, restraints were applied to O-H bonds. The position of the F atom was determined on the basis of (a) chemically reasonable position in the structure according to literature data, (b) electroneutrality of the structure, (c) difference map calculations, which clearly revealed the presence of water ligands, not OH bridges, between two Mn atoms, (d) critical examination of temperature factors, and, finally, (e) examination of the hydrogen bonding scheme. SHELXL-2018/3 software [41] was used for structure refinement and interpretation. Drawings of the structures were produced using ORTEPIII [42] and Mercury [43]. Details on crystal data, data collection, and structure refinement are given in Supplementary Materials in Table S1. All crystallographic details for both structures have also been deposited in the Cambridge Crystallographic Data Centre as deposition numbers 2435004 and 2435005, respectively. These data can be obtained free of charge via https://www.ccdc.cam.ac.uk/structures/ accessed on 12 April 2025 (or from the CCDC, 12 Union Road, Cambridge CB2 1EZ, UK; fax: +44 1223 336033; e-mail: deposit@ccdc.cam.ac.uk).

Nitrogen physisorption isotherms were recorded at −196 °C using the Autosorb iQ3 (Quantachrome, Boynton Beach, FL, USA). Before adsorption analysis, the samples were degassed under a vacuum for 10 h at 150 °C. The Brunauer–Emmett–Teller (BET) specific surface area was calculated from adsorption data in the relevant pressure range as determined using the Roquerol plot method. The t-plot method was used to determine micropore volume. Pore size distribution was determined using the enclosed software (ASIQwin 5.21) using the DFT pore size distribution function.

Solid-state magic angle spinning (MAS) nuclear magnetic resonance (NMR) spectra were recorded on a Bruker AVANCE NEO 400 MHz NMR spectrometer (Bruker, Billerica, MA, USA) equipped with a 4 mm CP-MAS probe. The Larmor frequencies of ^23^Na and ^19^F were 105.84 MHz and 376.51 MHz, respectively. Sample MAS frequencies were 15 kHz for the measurements. The ^23^Na spectra were acquired using a single π/2 pulse sequence with a duration of 1.5 μs, 1200 scans, and a delay between scans of 0.5 s. The ^19^F spectra were acquired using a Hahn echo pulse sequence of π/2 and π pulses with varying durations. In the case of the MnF_3_ spectra, 2.5 μs and 5.0 μs, respectively, 28 scans, and a delay between scans of 1 s was used. The ^19^F spectra of MnCIT-HF and MnCIT-NaF were measured with pulse lengths of 2.8 μs and 5.6 μs, respectively, 230 scans, and a delay between scans of 300 s. The shift axis in all spectra was referenced to an external reference of adamantane.

Thermogravimetric analysis (TGA) was performed on a TA Instruments Q5000 sample processor (TA Instruments, New Castle, DE, USA). The measurements were carried out in a continuous airflow (25 mL/min air), by heating samples from 25 °C to 650 °C at a rate of 10 °C/min.

The thermal behavior of the materials was also studied using high-temperature X-ray diffraction (HT XRD) in a vacuum on a PANalytical X’Pert PRO HTK (Malvern Panalytical, Malvern, UK) diffractometer (CuKα λ = 1.5406 Å radiation) in the 50–650 °C temperature range. The XRD patterns were collected using continuous scanning mode over a 2θ range of 5–50°, with a scanning speed of 0.013° and a counting time of 100 s per step.

## 4. Conclusions

The use of fluorine in the synthesis of manganese carboxylates revealed two new layered structures. In MnCit-HF, fluorine is incorporated in the structure at the bridging site between two manganese atoms, while in MnCit-NaF, fluorine acts as a tridentate ligand connecting three Mn polyhedra. Comparison with the crystal structures obtained from the same ratio of manganese and citric acid [30,31,34,35], but in a fluorine-free synthesis, revealed that the addition of HF significantly changed the crystallization mechanisms, which resulted in the new structures. The structure-directing role of HF was already observed in some inorganic porous materials [38] and was thus transferred to manganese citrates.

The obtained products, together with a fluorine-free Mn citrate, were tested as sacrificial precursors for mesoporous oxide and hierarchically porous carbon formation. In the case of K-MnCit-NaF and C-MnCit-HF, multi-phase products were obtained. All three sample-derived oxides showed some mesoporosity, with the MnCit-HF-derived oxide having the smallest pores and the MnCit-derived oxide having the largest pores. In the case of the derived carbons, the HF-free sample showed more microporosity, while the MnCit-HF-derived sample showed more mesoporosity, as evidenced by the higher total pore volume of the sample.

The data obtained confirmed that thermal treatment of the materials resulted in porous products that have further potential as catalysts, depending on the required micro-/meso-/macroporosity of the catalyst and the compatibility of the reaction with the added impurities, which require further studies.

## Data Availability

AIF files available upon request.

## Supplementary Materials

The following supporting information can be downloaded at: https://www.mdpi.com/article/10.3390/molecules30081794/s1, Section S1 (Tables S1–S5) with crystallographic data, References [44,45] are cited in the Supplementary Materials. Section S2 ssNMR spectra. Section S3 N_2_ isotherms and pore size distribution, TGA data, and Section S4 EDX mapping results for carbonized samples.
