# Supplementary material for: Impact of Fluorine in Manganese Citrate Synthesis on Structure and Value-Added Decomposition Products
Source: Molecules. 2025 Apr 16;30(8):1794. doi: 10.3390/molecules30081794 (PMC12029556; doi:10.3390/molecules30081794)
Supplement: Supplementary file 1 [file molecules-30-01794-s001.zip › molecules-3589372-supplementary.pdf]

molecules

MDPI

Supporting information

Impact of fluorine in manganese citrate synthesis on their structure and value-added decomposition products

Aljaž Škrjanc[1,2], Emanuela Trstenjak[1], Mojca Opresnik[1], Matej Gabrijelčič[1,3], Amalija Golobič[4] and Nataša Zabukovec Logar[1,2*]

[1] National Institute of Chemistry, Hajdrihova 19, SI-1000 Ljubljana, Slovenia; emanuela.trstenjak@silkem.si, aljaz.skrjanc@ki.si, mojca.opresnik@ki.si , matej.gabrijelcic@ki.si
[2] School of Science, University of Nova Gorica, Vipavska 13, SI-5000 Nova Gorica, Slovenia
[3] Faculty of Mathematics and Physics, University of Ljubljana, Jadranska ulica 19, SI-1000 Ljubljana, Slovenia
[4] Faculty of Chemistry and Chemical Technology, University of Ljubljana, Večna pot 113, SI-1000 Ljubljana, Slovenia; amalija.golobic@fkkt.uni-lj.si
* Correspondence: natasa.zabukovec@ki.si

Supporting information

Contents

S1. MnCit-HF and MnCit-NaF crystal structure data 2

S2. NMR characterization........ 11

S3. TGA and sorption analysis 13

S4. EDS mapping results 15

S1. MnCit-HF and MnCit-NaF crystal structure data

Table S1. Crystal data and structure refinement parameters for MnCit-HF and MnCit-NaF.

**Compound**	**MnCit-HF**	**MnCit-NaF**
Formula of compound	$[Mn_2(C_6H_5O_7)(H_2O)_2F]$	$Na(H_2O)_2[Mn_3(C_6H_5O_7)_2(H_2O)F]$
Formula relative weight	354.01	639.06
Crystal system	orthorhombic	monoclinic
Space group	$P2_12_12_1$	$P2_1/m$
a [Å]	6.9389(2)	6.5914(3)
b [Å]	10.0843(3)	14.1538(5)
c [Å]	14.5156(4)	10.5184(4)
α, β, γ [°]	90, 90, 90	90, 93.889(4), 90
V [Å^3]	1015.71(5)	979.04(7)
Z	4	2
D_c [mg m^{-3}]	2.315	2.168
Crystal size [mm^3]	0.18 x 0.14 x 0.12	0.30 x 0.18 x 0.03
Color, shape of crystals	Colorless, prism	Colorless, plate
T [K]	150.0(1)	150.0(1)
λ(MoK$_\alpha$) [Å]	0.71073	0.71073
Limiting indices	$-9 \leq h \leq 9$, $-14 \leq k \leq 14$, $-19 \leq l \leq 19$	$-9 \leq h \leq 9$, $-19 \leq k \leq 19$, $-14 \leq l \leq 13$
Θ_{max} [°]	30.44	30.45
R_{int}	0.0582	0.0305
μ(MoK$_\alpha$) [mm^{-1}]	2.543	2.028
Reflections collected	22812	19273
Independent reflections	2824	2789
Observed data [$F^2 \geq 2\sigma(F^2)$]	2693	2356
Parameters / restraints	183 / 4	179 / 5
Contributing reflections	2824	2789
R [F^2]	0.0249	0.0270
R_w [F^2]	0.0523	0.0675
Goodens of fit, S	1.0850	1.104
$\Delta\rho_{max}$ [Å^{-3}]	0.404	0.418
$\Delta\rho_{min}$ [Å^{-3}]	-0.396	-0.375

The Flack parameter for MnCit-HF is -0.017(9).

Table S2. Atomic coordinates and equivalent isotropic displacement parameters [Å^2] for MnCit-HF and MnCit-NaF.

$$U_{eq} = \frac{1}{3} a_i a_j \sum_i \sum_j U_{ij} a_i^* a_j^*$$

MnCit-HF	x	y	z	U_{iso}^* / U_{eq}
Mn1	1.39117 (6)	0.19381 (4)	0.74580 (3)	0.01086 (9)
Mn2	1.14918 (6)	−0.44117 (4)	0.65541 (2)	0.00960 (9)
F	1.5547 (2)	0.06628 (16)	0.82595 (10)	0.0133 (3)
O1	1.1344 (3)	0.06896 (17)	0.76453 (11)	0.0114 (3)
O2	1.0455 (3)	0.41471 (19)	0.54512 (13)	0.0146 (4)
O1W	1.6066 (3)	0.32392 (19)	0.68663 (13)	0.0148 (4)
O3	1.1899 (3)	0.32620 (18)	0.66379 (13)	0.0125 (4)
O2W	1.3348 (3)	0.3183 (2)	0.85877 (13)	0.0195 (4)
O4	0.8637 (3)	0.04199 (17)	0.68720 (12)	0.0126 (4)
O5	1.1317 (4)	−0.30236 (18)	0.54021 (12)	0.0185 (4)
O6	1.1430 (3)	−0.20539 (18)	0.67496 (12)	0.0135 (4)
O7	1.3526 (3)	0.07176 (19)	0.61887 (12)	0.0119 (4)
C1	1.1066 (4)	0.3132 (2)	0.58676 (16)	0.0106 (5)
C2	1.0791 (4)	0.1792 (2)	0.54268 (17)	0.0119 (5)
C3	1.1519 (4)	0.0590 (2)	0.59708 (15)	0.0095 (4)
C4	1.1144 (4)	−0.0661 (2)	0.53986 (16)	0.0124 (5)
C5	1.1343 (4)	−0.1972 (2)	0.58919 (16)	0.0113 (5)
C6	1.0430 (4)	0.0539 (2)	0.68908 (16)	0.0109 (4)
H1W1	1.562 (6)	0.411 (2)	0.674 (2)	0.038 (11)*
H1W2	1.273 (5)	0.400 (2)	0.846 (3)	0.038 (11)*
H2W1	1.716 (4)	0.331 (4)	0.725 (2)	0.035 (11)*
H2W2	1.343 (8)	0.302 (5)	0.9233 (11)	0.062 (15)*
H2A	1.145.031	0.179968	0.482185	0.014*
H2B	0.939821	0.166735	0.530804	0.014*
H4A	0.982174	−0.060431	0.514540	0.015*
H4B	1.204.544	−0.065928	0.487003	0.015*
H7	1.417 (6)	0.078 (4)	0.573 (2)	0.030 (10)*

MnCit-NaF	*x*	*y*	*z*	U_{iso}*/U_{eq}
Mn1	0.04522(6)	0.250000	0.24857(4)	0.00925(9)
Mn2	0.12783(4)	0.10846(2)	−0.02141(3)	0.00993(8)
Na	0.33692(19)	−0.250000	0.24439(12)	0.0248(3)
F	0.1020(2)	0.250000	0.04415(14)	0.0153(3)
O1	−0.1934(2)	0.14709(9)	0.22916(14)	0.0181(3)

O1w	0.0132(3)	0.250000	0.45174(18)	0.0145(4)
O2	−0.1191(2)	0.04573(8)	0.07706(12)	0.0132(3)
O2w	0.4032(4)	−0.250000	0.0230(2)	0.0284(5)
O3	−0.6318(2)	0.07175(10)	0.11598(13)	0.0180(3)
O3w	0.5578(4)	−0.250000	0.4384(2)	0.0323(5)
O4	−0.7497(3)	0.13747(11)	0.28836(14)	0.0290(4)
O5	0.0512(2)	−0.13181(9)	0.19051(13)	0.0176(3)
O6	0.1646(2)	−0.13093(9)	0.39224(13)	0.0154(3)
O7	−0.3762(2)	−0.08392(9)	0.16043(12)	0.0117(3)
C1	−0.1988(3)	0.06743(12)	0.17949(17)	0.0107(3)
C2	−0.3048(3)	−0.01185(11)	0.25002(16)	0.0092(3)
C3	−0.1526(3)	−0.05681(13)	0.34831(18)	0.0144(4)
C4	0.0340(3)	−0.10904(12)	0.30560(17)	0.0120(3)
C5	−0.4807(3)	0.02844(12)	0.31972(17)	0.0111(3)
C6	−0.6307(3)	0.08447(12)	0.23528(18)	0.0130(4)
H1w1	−0.066(4)	0.2986(14)	0.484(3)	0.047(8)*
H1w2	0.382(5)	−0.2969(17)	−0.039(2)	0.070*
H1w3	0.684(3)	−0.250000	0.489(3)	0.074(17)*
H2w3	0.456(4)	−0.250000	0.498(3)	0.070*
H3A	−0.102834	−0.006079	0.407276	0.017*
H3B	−0.229131	−0.101852	0.398854	0.017*
H5A	−0.553475	−0.024269	0.358555	0.013*
H5B	−0.425006	0.069702	0.389635	0.013*
H7	−0.478(4)	−0.0630(18)	0.126(2)	0.028(7)*

Table S3. Bond distances [Å] and angles [°] for MnCit-HF and MnCit-NaF.

**MnCit-HF**			
Mn1—F	2.0724 (15)	O1—C6	1.275 (3)
Mn1—O2W	2.102 (2)	O2—C1	1.262 (3)
Mn1—O1W	2.166 (2)	O3—C1	1.266 (3)
Mn1—O1	2.1986 (18)	O4—C6	1.250 (3)
Mn1—O7	2.2319 (18)	O5—C5	1.277 (3)
Mn1—O3	2.2693 (18)	O6—C5	1.249 (3)
Mn2—F^{i}	2.0741 (16)	O7—C3	1.434 (3)
Mn2—O5	2.1841 (19)	C1—C2	1.508 (3)
Mn2—O2^{ii}	2.2786 (19)	C2—C3	1.533 (3)
Mn2—O1^{iii}	2.2875 (18)	C3—C4	1.532 (3)
Mn2—O4^{iii}	2.2926 (17)	C3—C6	1.535 (3)
Mn2—O3^{ii}	2.3660 (18)	C4—C5	1.510 (3)
Mn2—O6	2.3949 (19)		
F—Mn1—O2W	91.97 (7)	O3^{ii}—Mn2—O6	168.65 (6)
F—Mn1—O1W	102.75 (7)	Mn1—F—Mn2^{iv}	129.65 (8)
O2W—Mn1—O1W	94.36 (8)	O4^{iii}—Mn2—O6	87.41 (6)
F—Mn1—O1	91.08 (7)	O1^{iii}—Mn2—O6	83.11 (7)
O2W—Mn1—O1	95.44 (8)	Mn1—O1—Mn2^{v}	141.76 (8)
O1W—Mn1—O1	162.74 (7)	C1—O2—Mn2^{vi}	94.29 (15)
F—Mn1—O7	100.77 (7)	C1—O3—Mn1	133.14 (17)
O2W—Mn1—O7	162.01 (8)	C1—O3—Mn2^{vi}	90.14 (15)
O1W—Mn1—O7	95.13 (7)	Mn1—O3—Mn2^{vi}	133.14 (8)
O1—Mn1—O7	71.88 (7)	C6—O4—Mn2^{v}	90.58 (15)
F—Mn1—O3	175.20 (7)	C5—O5—Mn2	96.05 (15)
O2W—Mn1—O3	86.76 (7)	C5—O6—Mn2	87.06 (15)
O1W—Mn1—O3	81.97 (7)	C3—O7—Mn1	110.38 (14)
O1—Mn1—O3	84.43 (7)	O2—C1—O3	119.5 (2)
O7—Mn1—O3	79.49 (7)	O2—C1—C2	118.8 (2)
F^{i}—Mn2—O5	97.57 (8)	O3—C1—C2	121.7 (2)
F^{i}—Mn2—O2^{ii}	115.31 (7)	C1—C2—C3	116.7 (2)
O5—Mn2—O2^{ii}	81.56 (7)	O7—C3—C4	111.0 (2)
F^{i}—Mn2—O1^{iii}	141.66 (6)	O7—C3—C2	111.3 (2)
O5—Mn2—O1^{iii}	108.21 (8)	C4—C3—C2	108.41 (19)
O2^{ii}—Mn2—O1^{iii}	96.54 (7)	O7—C3—C6	106.81 (18)
F^{i}—Mn2—O4^{iii}	84.92 (7)	C4—C3—C6	111.1 (2)
O5—Mn2—O4^{iii}	143.92 (7)	C2—C3—C6	108.2 (2)
O2^{ii}—Mn2—O4^{iii}	129.83 (7)	C5—C4—C3	116.61 (19)
O1^{iii}—Mn2—O4^{iii}	57.55 (6)	O6—C5—O5	120.0 (2)
F^{i}—Mn2—O3^{ii}	84.89 (7)	O2^{ii}—Mn2—O6	135.30 (7)

O5—Mn2—O3^{ii}	132.96 (7)	O6—C5—C4	122.4 (2)
O2^{ii}—Mn2—O3^{ii}	56.04 (6)	O5—C5—C4	117.5 (2)
O1^{iii}—Mn2—O3^{ii}	96.94 (7)	O4—C6—O1	121.7 (2)
O4^{iii}—Mn2—O3^{ii}	83.11 (6)	O4—C6—C3	118.3 (2)
F^{i}—Mn2—O6	88.08 (7)	O1—C6—C3	119.9 (2)
O5—Mn2—O6	56.87 (6)		

Symmetry codes: (i) −x+3, y−1/2, −z+3/2; (ii) x, y−1, z; (iii) −x+2, y−1/2, −z+3/2; (iv) −x+3, y+1/2, −z+3/2; (v) −x+2, y+1/2, −z+3/2; (vi) x, y+1, z

**MnCit-NaF**			
Mn1—O4^{i}	2.1125(14)	Na—O6^{v}	2.6053(16)
Mn1—O4^{ii}	2.1125(14)	Na—O6	2.6053(16)
Mn1—O1	2.1435(13)	Na—C4^{v}	2.925(2)
Mn1—O1^{iii}	2.1435(13)	Na—C4	2.925(2)
Mn1—O1w	2.1616(18)	O1—C1	1.242(2)
Mn1—F	2.2074(15)	O2—C1	1.268(2)
Mn2—O5^{iv}	2.0930(14)	O3—C6	1.267(2)
Mn2—F	2.1292(6)	O4—C6	1.244(2)
Mn2—O3^{ii}	2.1352(14)	O5—C4	1.265(2)
Mn2—O2	2.1769(12)	O6—C4	1.249(2)
Mn2—O2^{iv}	2.2593(12)	O7—C2	1.446(2)
Mn2—O7^{iv}	2.2945(13)	C1—C2	1.539(2)
Na—O2w	2.398(3)	C2—C5	1.524(2)
Na—O3w	2.425(3)	C2—C3	1.530(3)
Na—O5^{v}	2.5537(17)	C3—C4	1.529(2)
Na—O5	2.5537(17)	C5—C6	1.509(3)
O4^{i}—Mn1—O4^{ii}	97.87(10)	O3w—Na—C4^{v}	101.22(7)
O4^{i}—Mn1—O1	170.71(6)	O5^{v}—Na—C4^{v}	25.57(5)
O4^{ii}—Mn1—O1	87.83(6)	O5—Na—C4^{v}	89.52(6)
O4^{i}—Mn1—O1^{iii}	87.83(6)	O6^{v}—Na—C4^{v}	25.27(5)
O4^{ii}—Mn1—O1^{iii}	170.71(6)	O6—Na—C4^{v}	88.75(5)
O1—Mn1—O1^{iii}	85.62(8)	O2w—Na—C4	112.69(6)
O4^{i}—Mn1—O1w	84.73(5)	O3w—Na—C4	101.22(7)
O4^{ii}—Mn1—O1w	84.73(5)	O5^{v}—Na—C4	89.52(6)
O1—Mn1—O1w	88.51(5)	O5—Na—C4	25.57(5)
O1^{iii}—Mn1—O1w	88.51(5)	O6^{v}—Na—C4	88.75(5)
O4^{i}—Mn1—F	92.55(5)	O6—Na—C4	25.27(5)
O4^{ii}—Mn1—F	92.55(5)	C4^{v}—Na—C4	86.02(7)
O1—Mn1—F	94.53(5)	C1—O1—Mn1	131.06(12)
O1^{iii}—Mn1—F	94.54(5)	C1—O2—Mn2	132.20(11)
O1w—Mn1—F	175.85(7)	C1—O2—Mn2^{iv}	116.70(11)
O5^{iv}—Mn2—F	94.24(6)	Mn2—O2—Mn2^{iv}	106.11(5)
O5^{iv}—Mn2—O3^{ii}	164.45(5)	C6—O3—Mn2^{vi}	126.12(12)

F—Mn2—O3^{ii}	94.69(6)	C6—O4—Mn1^{vi}	141.24(13)
O5^{iv}—Mn2—O2	94.13(5)	C4—O5—Mn2^{iv}	133.72(12)
F—Mn2—O2	98.68(5)	C4—O5—Na	93.83(11)
O3^{ii}—Mn2—O2	97.08(5)	Mn2^{iv}—O5—Na	131.06(6)
O5^{iv}—Mn2—O2^{iv}	85.95(5)	C4—O6—Na	91.81(11)
F—Mn2—O2^{iv}	172.56(5)	C2—O7—Mn2^{iv}	107.89(10)
O3^{ii}—Mn2—O2^{iv}	86.84(5)	Mn2—F—Mn2^{iii}	140.39(7)
O2—Mn2—O2^{iv}	73.89(5)	Mn2—F—Mn1	109.76(4)
O5^{iv}—Mn2—O7^{iv}	82.41(5)	Mn2^{iii}—F—Mn1	109.76(4)
F—Mn2—O7^{iv}	115.15(5)	O1—C1—O2	125.13(16)
O3^{ii}—Mn2—O7^{iv}	82.27(5)	O1—C1—C2	117.40(15)
O2—Mn2—O7^{iv}	146.14(5)	O2—C1—C2	117.44(14)
O2^{iv}—Mn2—O7^{iv}	72.26(4)	O7—C2—C5	110.66(14)
O2w—Na—O3w	132.73(10)	O7—C2—C3	108.47(14)
O2w—Na—O5^{v}	87.97(7)	C5—C2—C3	108.43(14)
O3w—Na—O5^{v}	125.34(6)	O7—C2—C1	109.80(13)
O2w—Na—O5	87.97(7)	C5—C2—C1	110.13(14)
O3w—Na—O5	125.34(6)	C3—C2—C1	109.31(14)
O5^{v}—Na—O5	81.85(7)	C4—C3—C2	120.44(15)
O2w—Na—O6^{v}	134.03(5)	O6—C4—O5	122.20(17)
O3w—Na—O6^{v}	76.05(6)	O6—C4—C3	115.72(16)
O5^{v}—Na—O6^{v}	50.51(4)	O5—C4—C3	122.03(17)
O5—Na—O6^{v}	102.21(6)	O6—C4—Na	62.91(10)
O2w—Na—O6	134.03(5)	O5—C4—Na	60.59(10)
O3w—Na—O6	76.05(6)	C3—C4—Na	165.82(12)
O5^{v}—Na—O6	102.21(6)	C6—C5—C2	113.78(14)
O5—Na—O6	50.51(4)	O4—C6—O3	124.65(18)
O6^{v}—Na—O6	80.61(7)	O4—C6—C5	117.42(16)
O2w—Na—C4^{v}	112.69(6)	O3—C6—C5	117.87(15)

Symmetry codes:(i) x+1, −y+1/2, z;(ii) x+1, y, z;(iii) x, −y+1/2, z;(iv) −x, −y, −z;(v) x, −y−1/2, z;(vi) x−1, y, z.

Table S4. Hydrogen-bonding geometry [Å, °] for MnCit-HF and MnCit-NaF.

**MnCit-HF**	**Distances [Å]**			**Angles [°]**
D—H···*A*	*D*—H	H···*A*	*D*···*A*	*D*—H···*A*
O1W—H1W1···F^{i}	0.95 (1)	1.77 (2)	2.694 (3)	166 (4)
O1W—H2W1···O6^{i}	0.94 (1)	1.79 (2)	2.673 (3)	155 (3)
O2W—H1W2···O4^{ii}	0.95 (1)	1.78 (1)	2.727 (3)	172 (4)
O2W—H2W2···O5^{iii}	0.95 (1)	1.71 (2)	2.649 (3)	170 (5)
O7—H7···O2^{iv}	0.80 (4)	1.93 (4)	2.735 (3)	173 (4)

Symmetry codes: (i) −*x*+3, *y*+1/2, −*z*+3/2; (ii) −*x*+2, *y*+1/2, −*z*+3/2; (iii) −*x*+5/2, −*y*, *z*+1/2; (iv) *x*+1/2, −*y*+1/2, −*z*+1.

**MnCit-NaF**	**Distances [Å]**			**Angles [°]**
D—H···*A*	*D*—H	H···*A*	*D*···*A*	*D*—H···*A*
O1w—H1w1···O6^{i}	0.94(1)	1.80(2)	2.677(2)	155(3)
O2w—H1w2···O1^{ii}	0.94(1)	2.41(2)	3.252(3)	149(3)
O2w—H1w2···O3^{ii}	0.94(1)	2.65(3)	3.327(2)	130(3)
O3w—H1w3···O1w^{iii}	0.95(1)	2.05(1)	2.981(3)	164(3)
O7—H7···O3	0.80(3)	2.16(3)	2.793(2)	137(2)

Symmetry codes: (i) −*x*, *y*+1/2, −*z*+1;(ii) −*x*, *y*−1/2, −*z*;(iii) −*x*+1, −*y*, −*z*+1

Table S5. Bond valence sums for the manganese ions in MnCit-HF and MnCit-NaF. B=0.37 Å, $R_0(Mn^{II}$-O)=1.765, $R_0(Mn^{III}$-O)=1.732, $R_0(Mn^{IV}$-O)=1.750, $R_0(Mn^{II}$-F)=1.698, $R_0(Mn^{III}$-F)=1.660, $R_0(Mn^{IV}$-F)=1.710 Å [1,2]

MnCit-HF

bond	bond length, R (Å)	Mn^{II}	Mn^{III}	Mn^{IV}
Mn1–F	2,0724	0,36	0,33	0,38
Mn1–O2W	2,1020	0,40	0,37	0,39
Mn1–O1W	2,1660	0,34	0,31	0,32
Mn1–O1	2,1986	0,31	0,28	0,30
Mn1–O7	2,2319	0,28	0,26	0,27
Mn1–O3	2,2693	0,26	0,23	0,25
Sum	Σ exp((R_o-R)/B)	1,95	1,78	1,90
Mn2–F^{i}	2,0741	0,36	0,33	0,37
Mn2–O5	2,1841	0,32	0,29	0,31
Mn2– O2^{ii}	2,2786	0,25	0,23	0,24
Mn2–O1^{iii}	2,2875	0,24	0,22	0,23
Mn2–O4^{iii}	2,2926	0,24	0,22	0,23
Mn2–O3^{ii}	2,3660	0,20	0,18	0,19
Mn2–O6	2,3949	0,18	0,18	0,17
Sum	Σ exp((R_o-R)/B)	1,80	1,65	1,75

Symmetry codes: (i) −x+3, y−1/2, −z+3/2; (ii) x, y−1, z; (iii) −x+2, y−1/2, −z+3/2;

MnCit-NaF

bond	bond length, R (Å)	Mn^{II}	Mn^{III}	Mn^{IV}
Mn1—F	2,2074	0,25	0,23	0,26
Mn1—O4^{i}	2,1125	0,39	0,36	0,38
Mn1—O4^{ii}	2,1125	0,39	0,36	0,38
Mn1—O1	2,1435	0,36	0,33	0,35
Mn1—O1^{iii}	2,1435	0,36	0,33	0,35
Mn1—O1w	2,1616	0,34	0,31	0,33
Sum	Σ exp((R_o-R)/B)	2,10	1,91	2,03
Mn2—F	2,1292	0,31	0,28	0,32
Mn2—O5^{iv}	2,0930	0,41	0,38	0,4
Mn2—O3^{ii}	2,1352	0,37	0,34	0,35
Mn2—O2	2,1769	0,33	0,3	0,32
Mn2—O2^{iv}	2,2593	0,26	0,24	0,25
Mn2—O7^{iv}	2,2945	0,24	0,22	0,23
Sum	Σ exp((R_o-R)/B)	1,92	1,75	1,87

Symmetry codes:(i) x+1, −y+1/2, z;(ii) x+1, y, z;(iii) x, −y+1/2, z;(iv) −x, −y, −z;

1. Brown, I.D. Recent developments in the methods and applications of the bond valence model. *Chem. Rev.* **2009**, *109*, 6858–6919, doi:10.1021/CR900053K
2. (IUCr) Bond valence parameters Available online: https://www.iucr.org/resources/data/datasets/bond-valence-parameters (accessed on Apr 12, 2025).

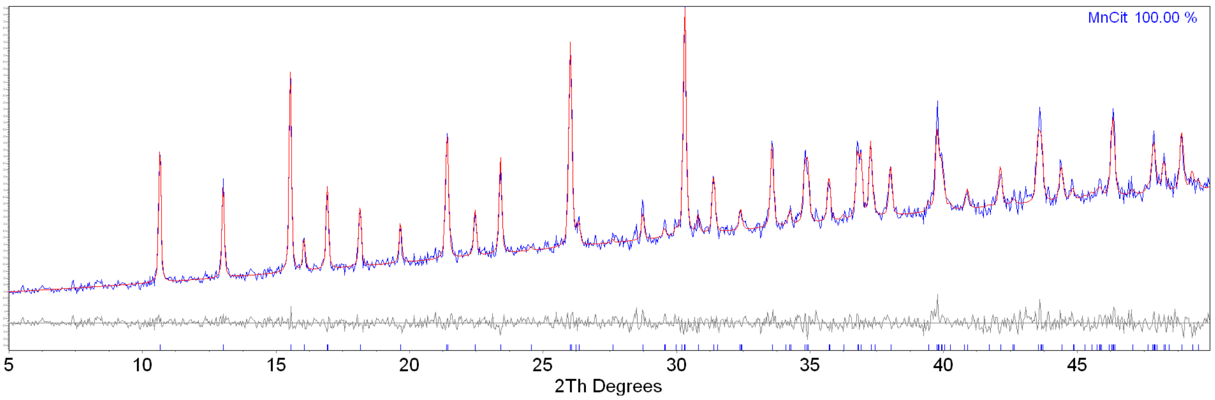

Figure S1. Rietveld refinement plot for single phase fluorine-free product, MnCit. Rwp: 5.57, GOF: 0.86.Red line represents calculated diffraction pattern of already known compound [Mn_2(HCit)(H_2O)] (MnCit) [33]. Vertical lines stand for its reflection positions. Blue color represents experimental and grey difference curve, respectively.

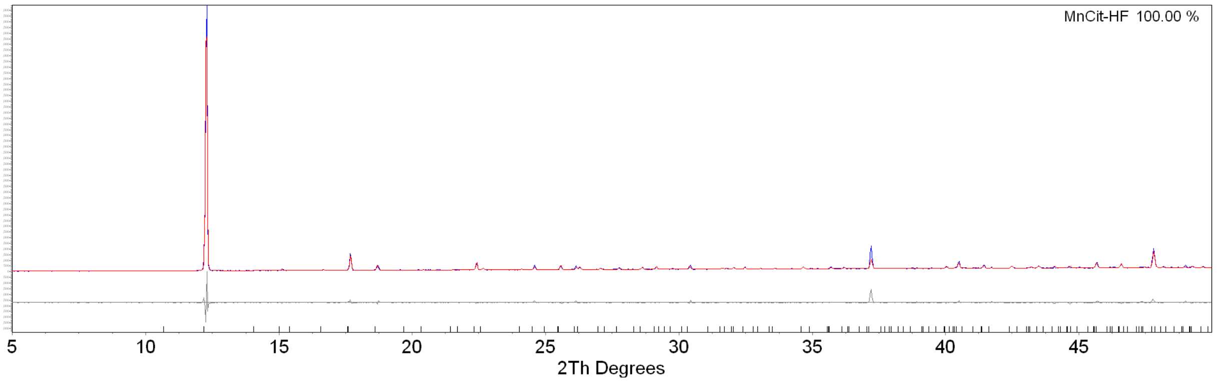

Figure S2. Rietveld refinement plot for single phase HF-based product, MnCit-HF. Rwp: 10.70,GOF: 1.79. Red line represents calculated diffraction pattern of compound [$Mn_2(C_6H_5O_7)(H_2O)_2F$]. Vertical lines stand for its reflection positions. Blue color represents experimental and grey difference curve, respectively.

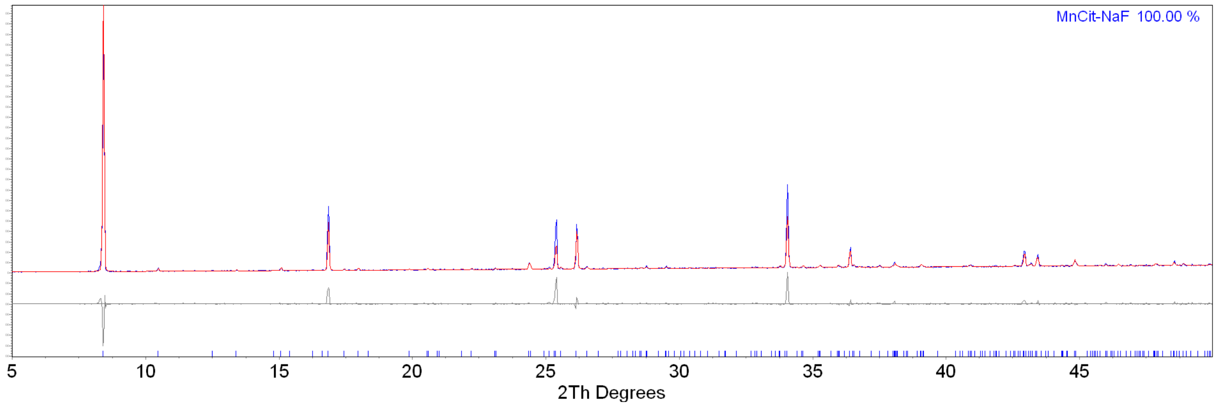

Figure S3. Rietveld refinement plot for single phase NaF-based product, MnCit-NaF. Rwp: 12.56, GOF: 2.07. Red line represents calculated diffraction pattern of compound $Na(H_2O)_2[Mn_3(C_6H_5O_7)_2(H_2O)F]$. Vertical lines stand for its reflection positions. Blue color represents experimental and grey difference curve, respectively.

S2. NMR characterization

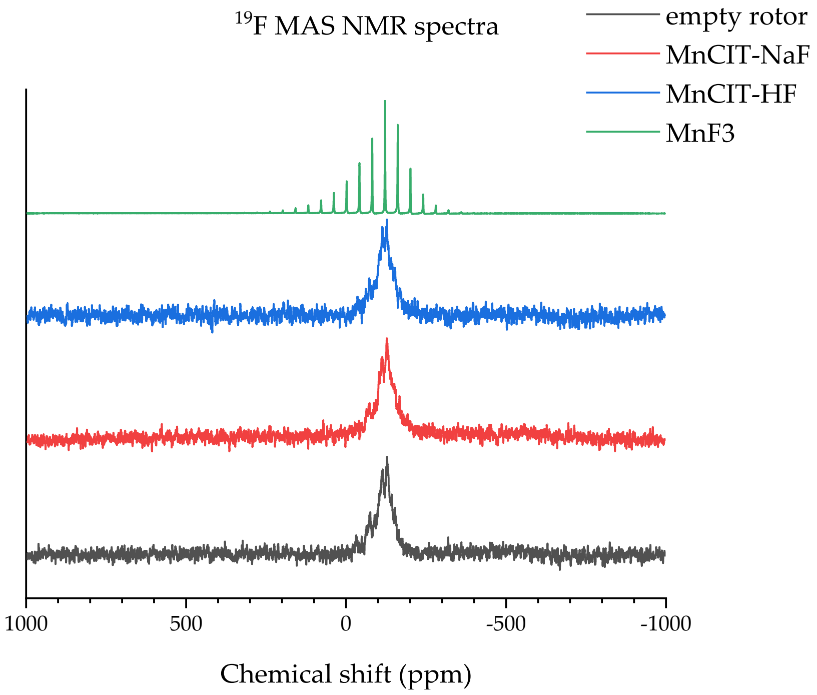

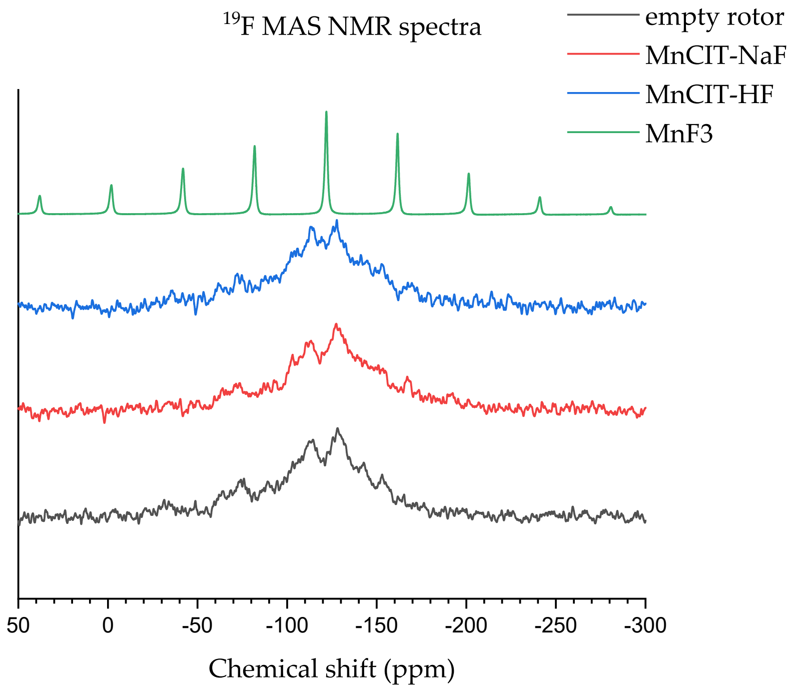

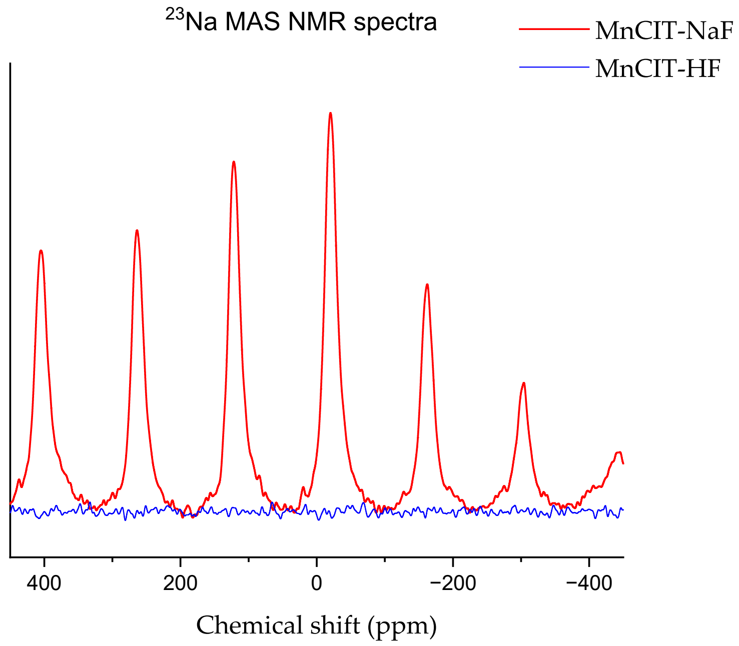

Figure S3. NMR SPECTRA OF PREPARED SAMPLES

S3. TGA and sorption analysis

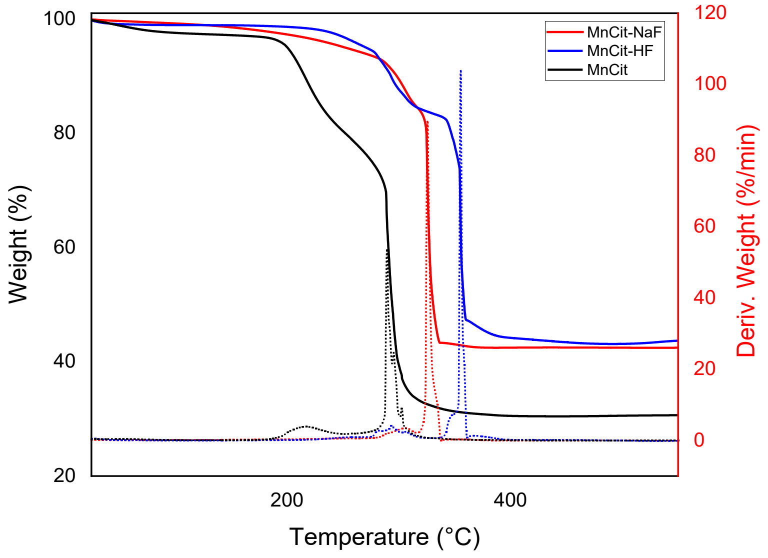

FIGURE S5: TGA OF PREPARED SAMPLES

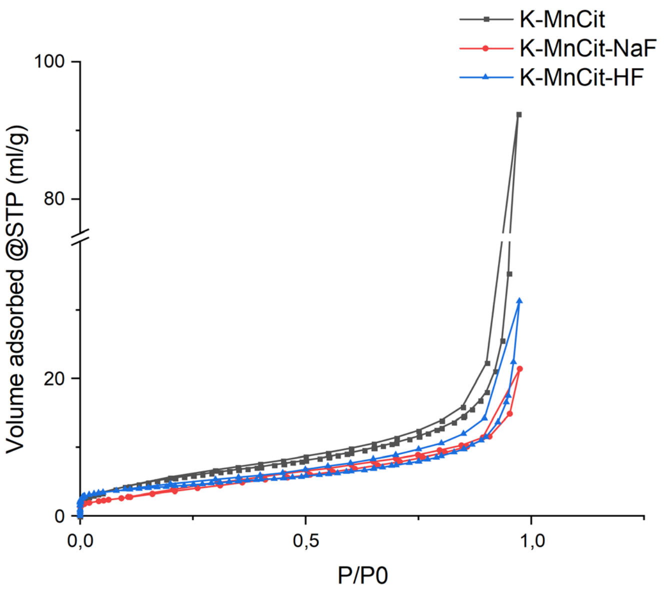

FIGURE S6: N_2 ISOTHERMS OF CALCINED SAMPLES

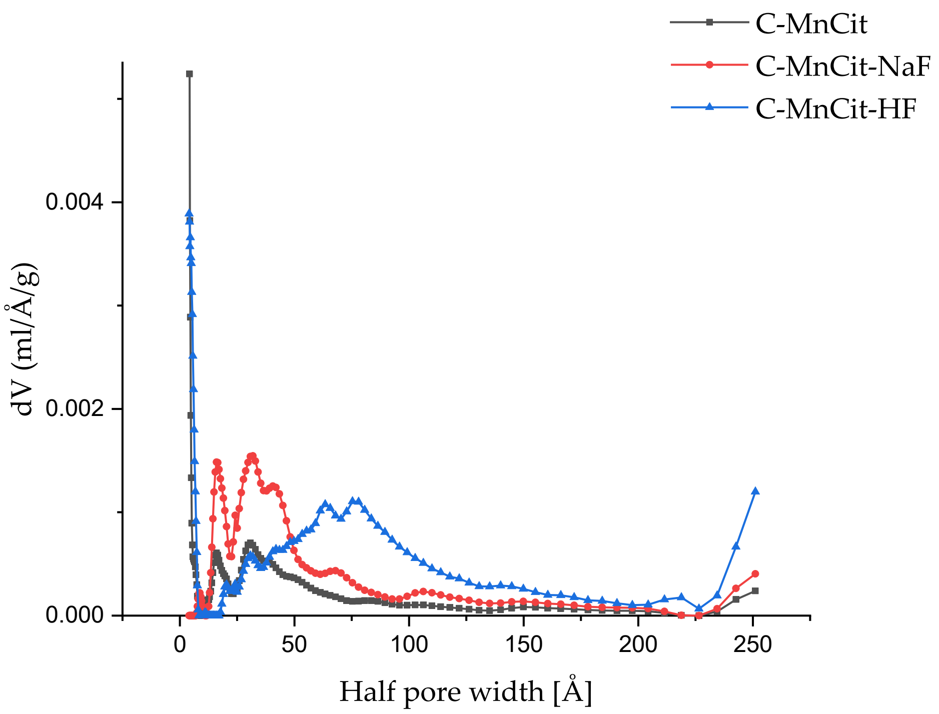

FIGURE S7: N_2 ISOTHERMS OF CARBONISED SAMPLES

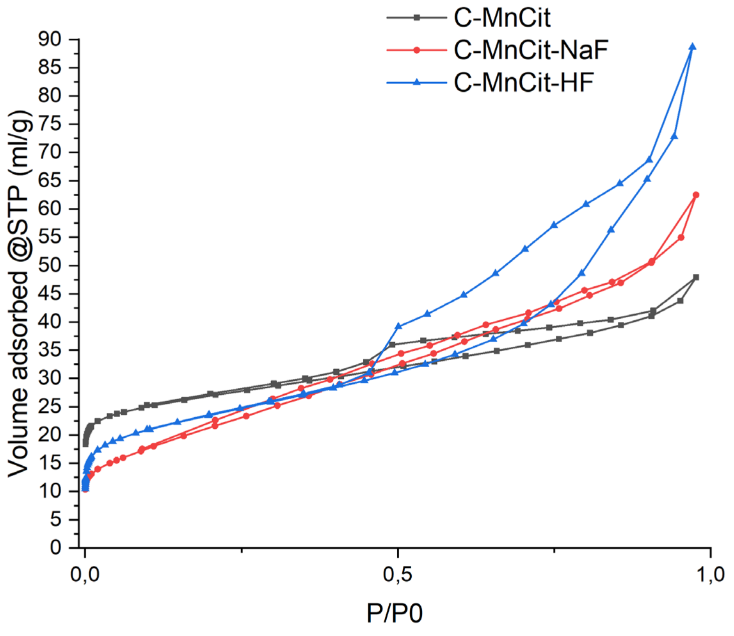

FIGURE S8: PORE SIZE DISTRIBUTION OF CARBONIZED SAMPLES

S4. EDS mapping results

1. C-MnCit:

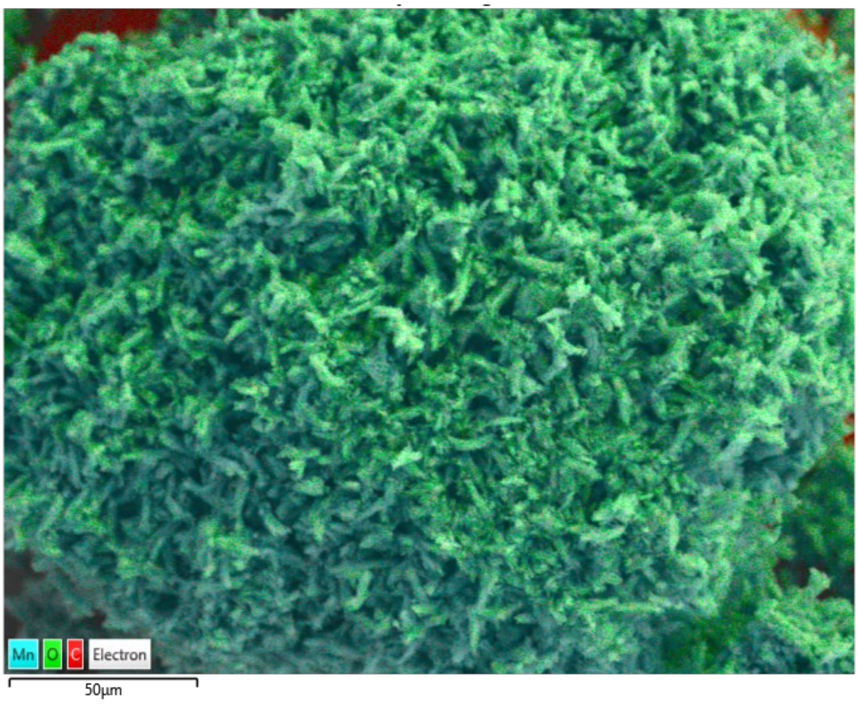

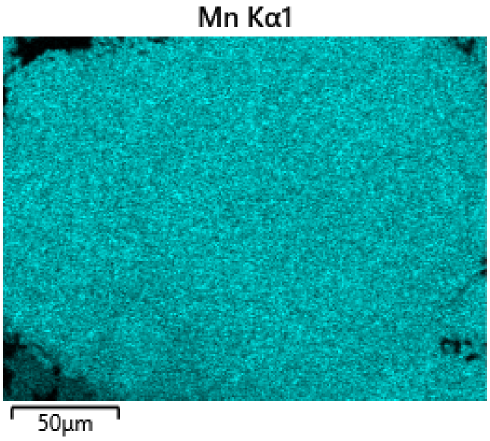

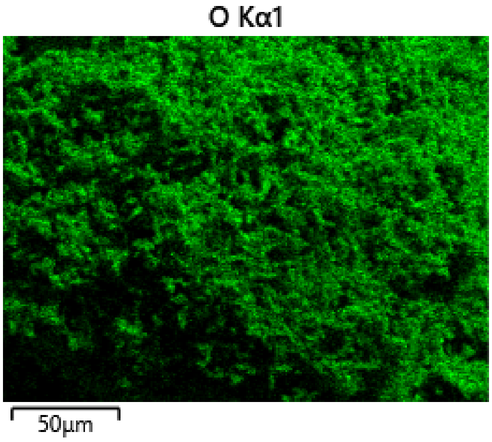

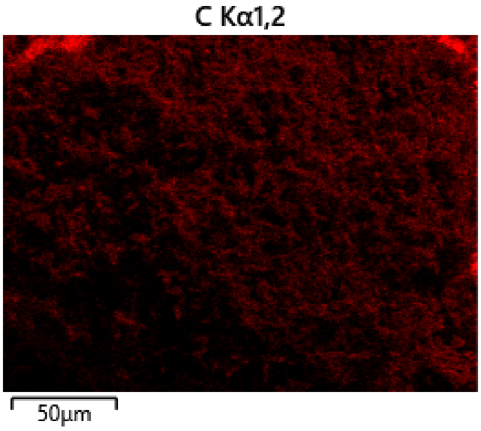

2. C-MnCit-HF

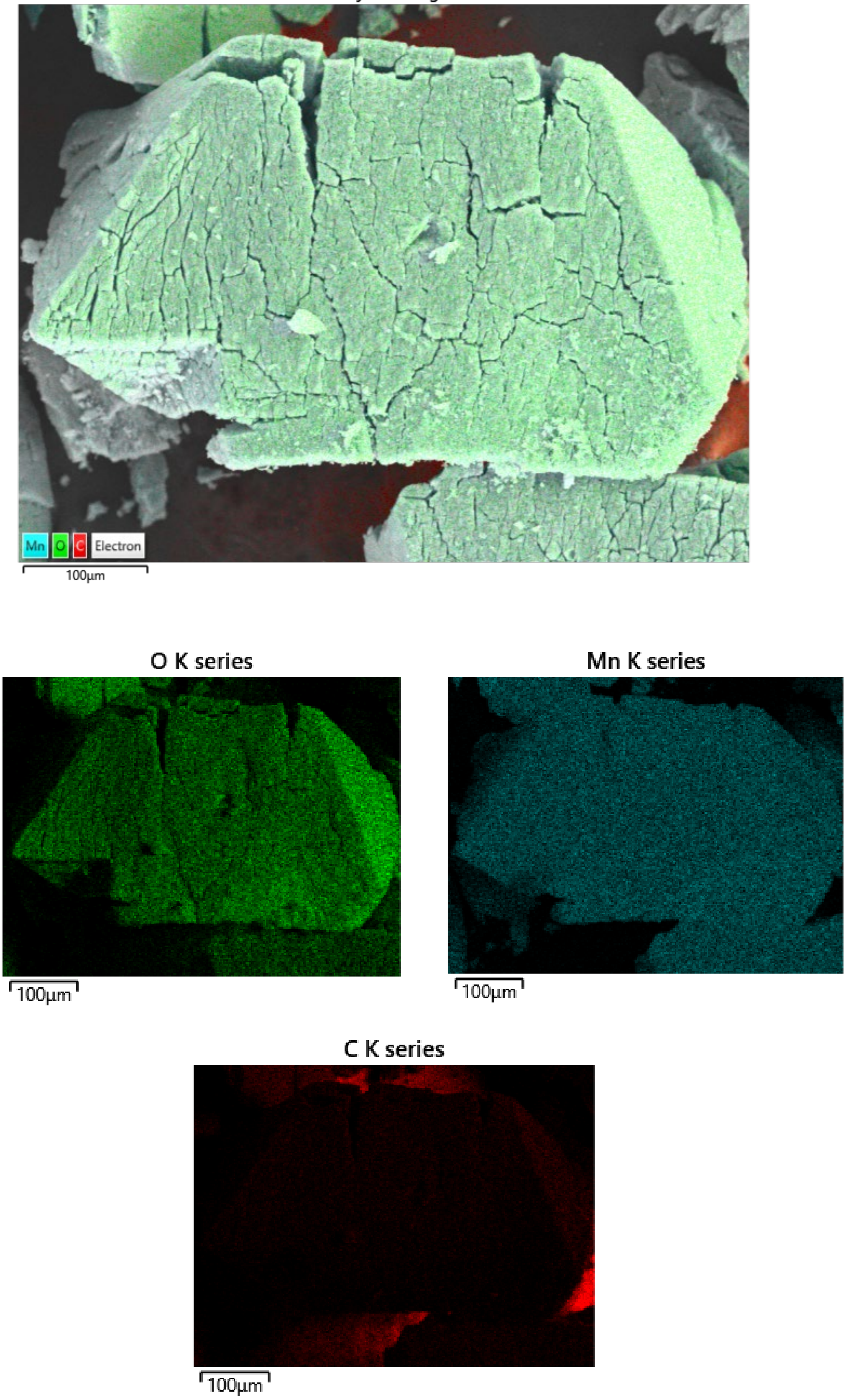
Mn
O
C
Electron
100µm
O K series
100µm
Mn K series
100µm
C K series
100µm

3. C-MnCit-NaF

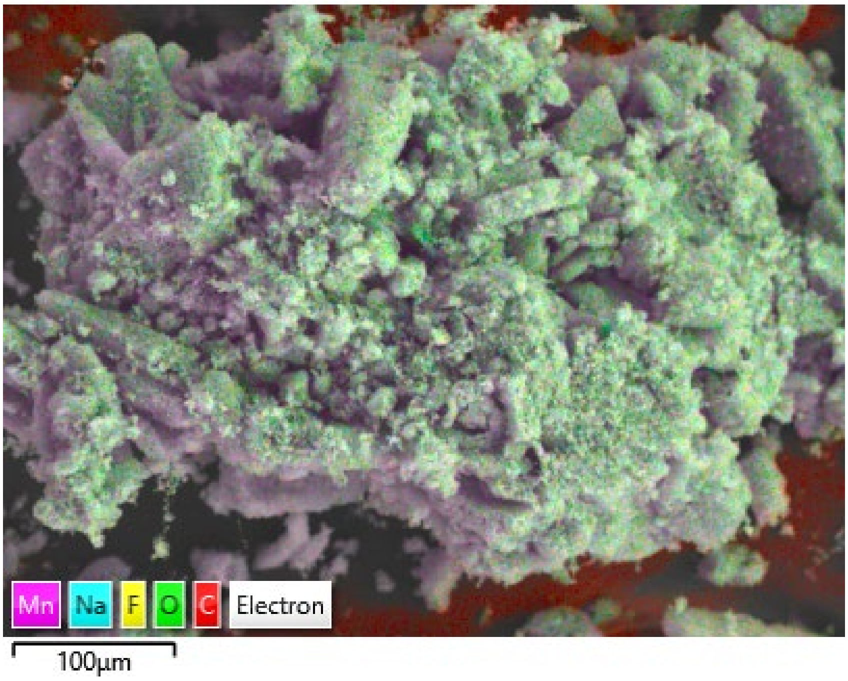

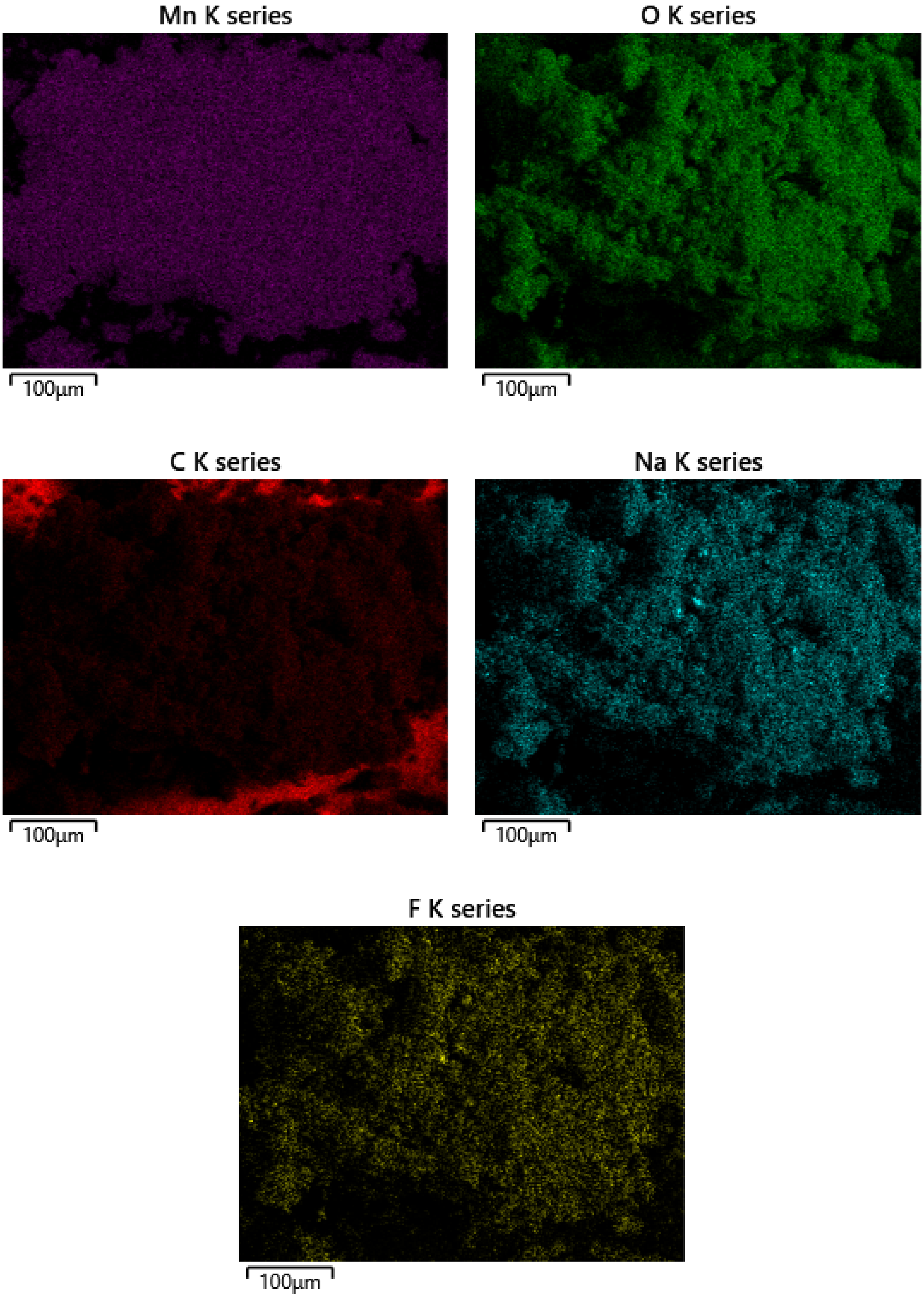
Mn K series
100µm
O K series
100µm
C K series
100µm
Na K series
100µm
F K series
100µm
